# CNN-LSTM vs. LSTM-CNN to Predict Power Flow Direction: A Case Study of the High-Voltage Subnet of Northeast Germany

**DOI:** 10.3390/s23020901

**Published:** 2023-01-12

**Authors:** Fachrizal Aksan, Yang Li, Vishnu Suresh, Przemysław Janik

**Affiliations:** 1 Faculty of Electrical Engineering, Wroclaw University of Science and Technology, 50-370 Wroclaw, Poland; 2 Department of Energy Distribution and High Voltage Engineering, Brandenburg University of Technology Cottbus-Senftenberg, 03046 Cottbus, Germany

**Keywords:** CNN-LSTM, LSTM-CNN, power flow prediction, network cluster

## Abstract

The massive installation of renewable energy sources together with energy storage in the power grid can lead to fluctuating energy consumption when there is a bi-directional power flow due to the surplus of electricity generation. To ensure the security and reliability of the power grid, high-quality bi-directional power flow prediction is required. However, predicting bi-directional power flow remains a challenge due to the ever-changing characteristics of power flow and the influence of weather on renewable power generation. To overcome these challenges, we present two of the most popular hybrid deep learning (HDL) models based on a combination of a convolutional neural network (CNN) and long-term memory (LSTM) to predict the power flow in the investigated network cluster. In our approach, the models CNN-LSTM and LSTM-CNN were trained with two different datasets in terms of size and included parameters. The aim was to see whether the size of the dataset and the additional weather data can affect the performance of the proposed model to predict power flow. The result shows that both proposed models can achieve a small error under certain conditions. While the size and parameters of the dataset can affect the training time and accuracy of the HDL model.

## 1. Introduction

Energy is the most important element for global economic growth [1]. However, to meet the needs of the global market, mainly fossil fuels are used. With the depletion of fossil fuel supplies and the increasing demand for electrical energy, we are now massively turning to renewable energy sources as an alternative to provide a more economical and diversified energy mix that ensures energy security and sustainability [2]. The enormous expansion and installation of renewable energy sources, such as wind turbines and photovoltaics (PV), in electrical grids can lead to a change in the power flow and also affect the extent of the bi-directional power flow via a transformer to or from the power grid system [3,4]. Furthermore, this phenomenon has also led to a significant change in the traditional power supply mechanisms, from a centralised to a decentralised power system and from a directional to a bi-directional power supply system [4]. In the past, the electricity supply mechanism operated by generating electricity in large central power plants on the high-voltage grid, which was then transmitted to end users through the transmission and distribution system. Therefore, power flows on the generation and consumption side were much more predictable. This is due to the advance planning of power plant deployment and the standardized creation of load profiles [3]. However, most renewables work in the opposite way, they can be installed together with energy storage at all grid levels, but mainly at the medium and lower voltage levels of the distribution grid system, so that integration becomes easier.

Therefore, the simple and decentralised integration of energy resources into the power system can bring new challenges. These include the challenge of predicting power flow due to the intermittent nature of renewables and also the occurrence of bi-directional power flows in the power system [5]. Bi-directional power flows have two opposite directions like tides. They can occur, for example, when renewable electricity generators become common for domestic use and a surplus of electricity generated by this generator is passed on to an electricity utility. In this case, the household electricity grid system will have a bi-directional power flow. Another challenge is the difficulty of predicting consumption behaviour with standardised load profiles. This is due to the numerous integrations of energy storage and e-mobility into the power grid [3], so that several end users have different types of loads, which are mostly inductive or capacitive.

Knowing the power flow in the grid is very important to know how much electricity is imported when the regional load is high and how much surplus power generation is exported from the investigated region. This can also help the distribution utilities to control the generated electricity that is transmitted to the load centres and distributed from there to the end-users. Furthermore, it can also help grid operators avoid congestion and manage the exchange of energy between the different connected electricity suppliers. Knowledge of the actual conditions and power flow on the grid is indeed important, both in the present and future state. However, all the above challenges in integrating renewable energy have led to unpredictable power flows in the grid, especially at transformers between voltage levels. Therefore, this study investigates the prediction of the bi-direction of power flow to meet these grid operation strategies.

There are several ways to make an accurate prediction. Currently, the development of machine learning (ML) and deep learning (DL) is increasing with the advances in the field of computer science. Therefore, it is not surprising that the methods using ML and DL are already used for all prediction and forecasting tasks in modern power systems [6,7,8,9]. The competitiveness of DL as a robust method capable of solving complex problems with a lower error rate has been demonstrated in reference [10]. In general, deep learning can work as a single network or as a hybrid network. A study in reference [11] examined several research works on load forecasting using different techniques, and one of the results shows that the hybrid method has higher accuracy compared to single model algorithms. Apart from the advantages, the DL method still has the challenge of finding the optimal configuration for the structure of the models.

To predict the direction of power flow using the DL method, the DL method must learn from historical power flow measurement data. However, the power flow can also be influenced by grid topologies and fluctuation generation, as renewable energies are dependent on the weather condition. Therefore, it is necessary to include weather data in the prediction of bi-directional power flow. In this paper, we present an approach to predict bi-directional power flow that takes into account power measurement data and weather data. The proposed prediction model is based on a hybrid deep learning (HDL) model using a convolutional neural network (CNN) and a long-term memory (LSTM) as the backbone. An LSTM is a special model that is usually used for time series predictions [12,13,14,15,16,17], while a CNN network is mainly used for processing images. However, this model is still suitable for time series prediction [18,19,20,21]. To answer the question of whether the HDL model is suitable for predicting the direction of power flow, we compare the performance of the CNN-LSTM model with the LSTM-CNN model to predict the direction of power flow in the single feeder of the power grid under study. In general, the contribution of our paper is summarized as follows:

- We present and analyse a hybrid deep learning model for predicting the direction of power flow in a single feeder of the power grid.
- We compare and evaluate the performance of the proposed HDL model (CNN-LSTM versus LSTM-CNN) with baseline models (CNN only and LSTM only) for power flow prediction.
- We investigate how weather data can influence the prediction result to determine the direction of power flow in the studied power system.
- We investigate how the HDL model behaves when the input data used has a different size and different parameters.

The rest of the paper is organised as follows: related work is presented in Section 2, a brief description of deep learning model structures is given in Section 3, Section 4 gives a basic description of the proposed methodology, Section 5 describes our case study and the dataset used, Section 6 presents the results and discussion, and the last section summarises the conclusions of this paper.

## 2. Related Work

Few scientific papers have been published in the field of bi-directional power flow prediction. However, there are some research papers dealing with power flow prediction that have similarities with our work. In reference [3], the authors used an LSTM network for vertical power flow forecasting at a transformer located between the medium- and high-voltage networks. The authors used an updating process where models that were trained regularly were checked. The experimental results showed that the proposed approach achieved a good improvement compared to the approach without an updating process. The study in reference [22] presents adaptive power flow prediction. The authors used a machine learning model to predict the voltages of the terminal nodes on the high- and low-voltage sides of the distribution network without knowing the line topology or impedance. The proposed model was comparable to the conventional impedance estimation from power flow analysis. In the study [23], the prediction of power flow was carried out using an artificial neural network (ANN). The main objective of the authors was to reduce the maximum prediction error of the model in predicting the magnitude of the bus voltage and the line load by applying appropriate data pre-processing techniques. A power flow analysis can be performed to learn more about the bi-directional power flow in an integrated power system with other power resources. Knowledge of bi-directional power flow is very important for power system operators to plan, maintain and modify circuits for facilities or loads that may require power during peak load or feed surplus power to the main power supply. In our study case, we performed two HDL models: CNN-LSTM and LSTM-CNN, to predict the direction of power flow on individual feeder lines of the power grid under study.

CNN-LSTM and LSTM-CNN are well-known models used to solve prediction and forecasting tasks in the field of power systems. For example, in reference [24], the proposed model CNN-LSTM was used to predict the hourly heating load. The authors employed the CNN to extract the spatial features and influencing factors of the heating load data, while the LSTM was used as a temporal feature extractor to extract the time lag features of the heating load data. Therefore, the proposed model is more suitable for predicting the heating load in the presence of nonlinearity and significant thermal inertia delay. Another study using the CNN-LSTM architecture can be found in reference [25]. In this work, a hybrid model was proposed to predict the short-term photovoltaic electricity generation. Based on the simulation results, it was shown that CNN-LSTM has excellent performance in terms of stability, accuracy and prediction compared to the standard algorithm ML and the single model DL.

If CNN-LSTM is proven to have good capabilities, then so is LSTM-CNN. A study in reference [26] proved that LSTM-CNN can achieve excellent results in load forecasting. The authors used the proposed model to predict the load of the next time step on different datasets. The result showed that the proposed model still has high accuracy. In a similar case of load prediction, LSTM-CNN was also used in reference [27]. In this study, LSTM was used to increase the sensitivity of the model and enhance the influence of important information in the features, while CNN was added to improve the model’s ability to perceive the sensitivity to data. In this way, the model achieved an excellent predictive performance. The summary of the research that deals with power flow and the evidence that CNN-LSTM and LSTM-CNN are good for prediction is presented in Table 1.

## 3. Deep Learning Model Structures

In this section, we present a brief description of the proposed HDL model for predicting the direction of power flow in a single feeder of the studied network cluster. Since the CNN-LSTM and LSTM-CNN models have been proposed in this paper. It is necessary to briefly discuss the LSTM and CNN networks. The reason is that these two individual models form the backbone of the proposed HDL model.

### 3.1. Long Short-Term Memory (LSTM) Network

The LSTM is a special type of recurrent neural network (RNN) first introduced by Hochreiter and Schmidhuber [28]. This model was developed to circumvent the problem of long-term dependence and to solve the vanishing gradient problem, since the standard RNN model is not able to learn long-term dependence. Therefore, this model includes memory cells and gates to regulate the network’s information and remember information over long periods of time. Indeed, the LSTM is a popular DL model used for all cases of forecasting and prediction. In a study in reference [29], the LSTM network showed good performance in predicting network losses in a Finnish power grid. Moreover, it can perform very well even without prior knowledge of the power system. Another study using LSTM can be found in reference [30]. The authors implement the proposed LSTM model to forecast hourly day-ahead solar irradiance based on weather forecast data. The proposed model performs slightly better than some competing algorithms because it takes into account the dependencies between different hours of the same day.

**Figure 1:**
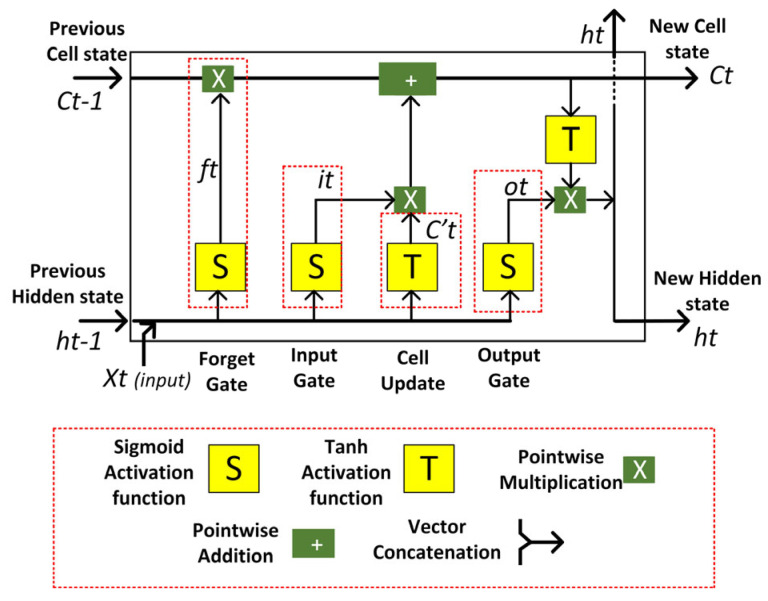
The architecture of LSTM network.

In general, an LSTM network consists of memory blocks called cells. Each cell has two states: the cell state and the hidden state. The cells in the LSTM network are used to make important decisions by storing or ignoring information about important components. These components are called gates, which are organised as follows: forget gates, input gates, and output gates. According to the structures shown in Figure 1, the LSTM model operates in three stages: In the first stage, the network works with the forget gate to check what kind of information needs to be ignored or stored for the cell state. The calculation starts by considering the input at the current time step (*x_t_*) and the previous value of the hidden state (*h*_(*t*−1)_) using the sigmoid function (*S*). The formula for the calculation in forget gate is as follows.

(1) $$f_{t}=S\left(wf\;\cdot \;\left[h_{\left(t-1\right)},x_{t}\right]+b_{f}\right)$$

In the second phase, the calculation of the network continues by converting the old cell state (*C*_(*t*−1)_) into a new cell state (*C_t_*). This process selects which new information must be included in the long-term memory (cell state). To obtain the new cell state value, the calculation process should take into account the reference value from the forgetting gate, the input gate and the cell update gate value. The formulas for this step are shown below.

(2) $$i_{t}=S\left(wi\;\cdot \;\left[h_{\left(t-1\right)},x_{t}\right]+b_{i}\right)$$

(3) $${C^{\prime }}_{t}=T\left(wc\;\cdot \;\left[h_{\left(t-1\right)},x_{t}\right]+b_{c}\right)$$

(4) $$C_{t}=\left(C_{\left(t-1\right)}\;\cdot \;f_{t}\right)+\left(i_{t}\;\cdot \;{C^{\prime }}_{t}\;\right)$$

Once the cell status update is complete, the final step is to determine the value of the hidden state (*h*_(*t*)_). The aim of this process is for the hidden state to act as the network’s memory, containing information about previous data and used for predictions. To determine the value of the hidden state, the calculation must have the reference value of the new cell state and the output gate (*o_t_*). The formula for this process is shown below.

(5) $$o_{t}=S\left(wo\;\cdot \;\left[h_{\left(t-1\right)},x_{t}\right]+b_{o}\right)$$

(6) $$h_{t}=o_{t}\;\cdot \;T\left(C_{t}\right)$$

### 3.2. Convolutional Neural Network (CNN)

Another variant of the deep learning model is the CNN network. This model has the ability to learn highly abstracted features of objects [31]. Therefore, it is very suitable for visual image analysis and recognition [6,32,33]. However, the CNN model also has a layer that is able to learn the features of sequence data with multiple variables. Therefore, it can also be used for any prediction task. In the case of fault detection, reference [34] shows that CNN has been used with attentive density to detect and identify the fault types and severities of rolling bearings. In [35], the CNN architecture was used to solve the problem of blade icing by analysing the imbalance of the supervisory control and data acquisition (SCADA) data of a wind turbine.

**Figure 2:**
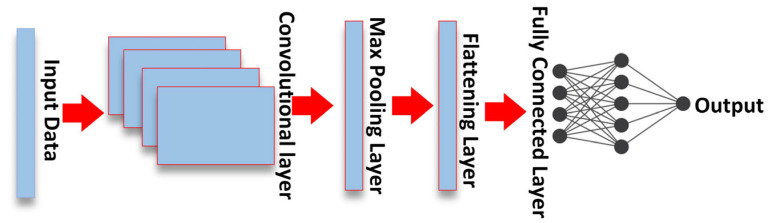
The structure of CNN network.

According to reference [19], a typical CNN model, as shown in Figure 2, comprises several layers: convolutional layer, pooling layer, a flattening layer, and a fully connected layer. The convolutional layer is the main component of the CNN network, which operates on the principle of sliding windows and weight sharing to reduce computational complexity. In this layer, the kernel method is used to extract various features from the input data. The next layer is the pooling layer. This layer is designed to reduce the size of the feature map involved by reducing the connections between layers and running each feature map independently. The main goal of the pooling operation is to reduce the dimensionality and extract the dominant features for efficient training of the model [6]. There are several types of pooling operations: max pooling and average pooling. Before proceeding with the fully connected linked layer (FC), it is necessary to use the flattening layer to create a one-dimensional vector, because the FC layer consists of the weights and biases along with the neurons to connect the neurons between the different layers. The FC layer is sometimes inserted as the last layer before the output layer of the CNN network.

### 3.3. Hybrid Deep Learning Model

**Figure 3:**
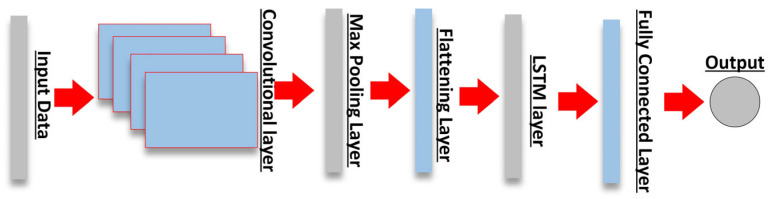
The structure of CNN-LSTM model.

As previously stated, two types of HDL were used for training directional power flow prediction. They are the models CNN-LSTM and LSTM-CNN. The structure of the two HDL models used in this paper is shown in the following Table 2. The architecture of CNN-LSTM (see Figure 3) was developed with CNN layers on the front end. The aim is to extract the features of the input dataset. The outputs of the CNN layers were then passed to the LSTM layers and a dense layer at the output to support sequence prediction.

**Figure 4:**
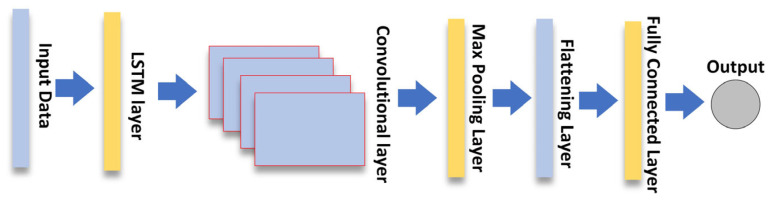
The structure of LSTM-CNN model.

On the other hand, the structure of LSTM-CNN (see Figure 4) is in a different sequence. The LSTM layers were used to order the sequence of time series data as input. The idea behind this is that the output of the LSTM layers contains more new information, which is then fed into the CNN layers to extract local features. The output of this convolutional layer is then pooled into a smaller dimension and passed to the dense layer to predict the final output.

## 4. Methodology

**Figure 5:**
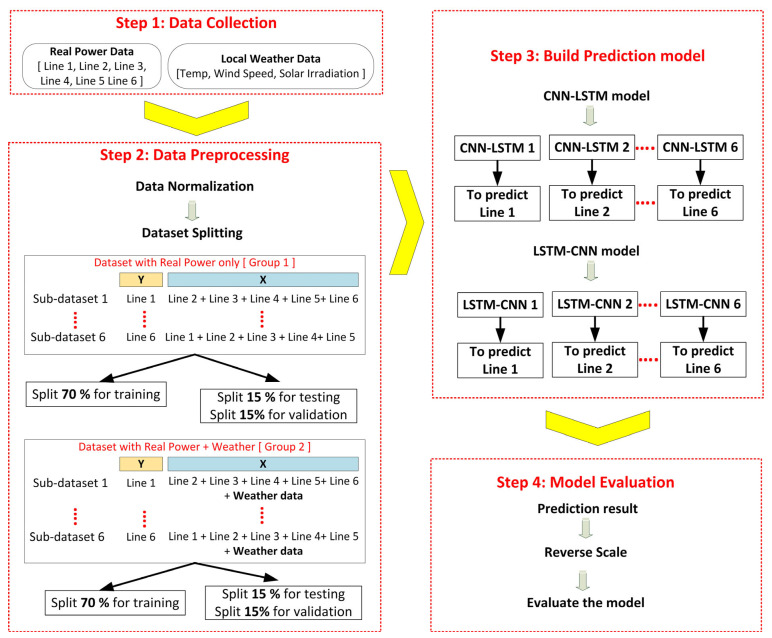
Proposed methodology.

To compare the performance of CNN-LSTM and LSTM-CNN in predicting the direction of load flow of each line in the power grid, the proposed methodology is shown in Figure 5. The proposed approach is divided into four steps: data collection, pre-processing of the data, creation of the models CNN-LSTM and LSTM-CNN and evaluation of the models.

### 4.1. Step 1: Data Collection

Data collection is a crucial step because all further steps depend on the availability of the data. Data collection is about gathering all the necessary data from the available sources. It is important to clean and filter the data before it is used. In this work, the raw data for directional power flow is collected from a power grid under study and the weather data is obtained from a weather service provider. Therefore, the quality of the data used in this work is suitable for training and testing the proposed hybrid deep learning model for predicting the direction of power flow.

### 4.2. Step 2: Data Pre-Processing

After data collection, the next step is to pre-process the data. The main objective of this phase is to prepare and convert the raw data into a format suitable for the HDL model. The implementation of data pre-processing is very important for any type of deep learning model as it can improve the model accuracy by improving the quality of the data and extracting valuable information from the data [36]. In this work, various data pre-processing techniques were used, ranging from normalising the data to splitting the dataset.

#### 4.2.1. Data Normalization

The datasets used in this study come from different sources, and their parameters have different units and scales. These differences in the datasets may affect the performance of HDL during the learning process and, even worse, increase the generalisation error. Therefore, to avoid this problem, it was necessary to scale or normalise all variables in the dataset. Moreover, this can also improve the performance of HDL as all input variables are scaled to a standard range [37]. In this study, the numerical scaling method min–max normalisation was used. The formula for converting the original value into a normalised value is shown in the following equation.

(7) $$x^{\prime }=\frac{x-min\left(x\right)}{max\left(x\right)-min\left(x\right)}$$

where *x*′ is the normalised value, *x* is the original value, *max*(*x*) is the maximum value of *x* and *min*(*x*) is the minimum value of *x*.

#### 4.2.2. Dataset Splitting

For the development and evaluation of a predictive model. Sometimes the input data must be prepared in a suitable way and divided into a training, a validation and a test dataset. In principle, there is no optimal percentage for the splitting ratio. However, there are several ways to split the dataset, e.g., 90% for training and 10% for testing [38,39], or 80% for training and 20% for testing [40,41]. However, this study refers to the scenario of 70% for the training dataset, 15% for the validation dataset and 15% for the test dataset, based on references [15,42,43,44,45]. The training and validation dataset was split using the train test split library of the scikit learn framework. In splitting the data, we performed a control shuffle of the data with a random state value of 42.

Before dividing the dataset, we categorised two datasets by size and parameters in the proposed methodology. The aim of this approach was to follow our research contribution to find out the extent to which weather data can influence the proposed HDL model to predict the direction of power flow at each line in the power grid under study. Therefore, the first group of datasets contained only directional power flow data, while the dataset of the second group contained directional power flow and weather data.

### 4.3. Step 3: Build Prediction Models

Our proposed work focuses on predicting the direction of power flow in each line of the power system under study using two types of HDL models. The first model is the CNN-LSTM and the second model is the LSTM-CNN. Both HDL models use the same two individual networks of CNN and LSTM. However, they are just constructed in a different order. When building an HDL model, there is no direct information about the optimal model architecture for a particular model. For example, how many hidden layers, activation functions and optimisers should be used. Therefore, it is necessary to select an optimal set of parameter configurations, as these parameters are used to control and manage the learning process of the HDL model to accurately predict the output. There are different ways to tune the hyperparameters. In this study, we randomly selected the parameters to tune the hyperparameters of the proposed HDL models and the baseline models (CNN only and LSTM only).

In this work, the CNN-LSTM architecture integrated several layers. In the first layer of the model, there are two stacks of convolutional layers with the activation function rectified linear unit (relu). This is followed by the max-pooling layer and the flattening layer. After this, the stack LSTM layer with the activation function relu was built. A dense layer is connected as the output layer. This layer uses the activation function leaky rectified linear unit (LeakyRelu). As the optimiser, we choose Adam with a learning rate of 0.01 and a loss function with mean square error.

On the other hand, for the LSTM-CNN architecture. We used the same layers and activation functions as in the CNN-LSTM model. The only difference is the order of the layers. The model LSTM-CNN was built from stacks of the LSTM network, which were then passed to stacks of convolutional layers, followed by a sequence of max-pooling layers and the flattening layer. In the output there is a dense layer. The optimiser and loss function implemented in the LSTM-CNN model are the same as in the CNN-LSTM model. A summary of the architectures of CNN-LSTM, LSTM-CNN and the baseline models can be found in Table 2.

There are several important hyperparameters that need to be set in the proposed HDL and baseline models, such as the number of batch sizes and the number of epochs. The batch size is a parameter that specifies the number of samples that are run before the model parameters are updated. Epoch number is a hyperparameter that specifies how often the learning algorithm is applied to the training dataset. Using too few or too many epochs may result in under-fitting or over-fitting. In this work, for each proposed HDL model and baseline model during the training process, we set the same batch size with a value of 32 and the same number of epochs with a value of 25. All structures and layers of all models were created using TensorFlow and the Keras library. During the experimental research, the proposed HDL along with baseline models were trained and tested on a laptop with the technical specifications listed in Table 3.

### 4.4. Step 4: Evaluate the Proposed HDL Model

Model evaluation is a very important step to assess and measure the performance and accuracy of the proposed HDL model using the metric scores. The evaluation metrics used for this study were selected based on the recommendations of studies and reports in the field of predictive cases. The metrics are the root mean square error (*RMSE*), the mean absolute error (*MAE*) and the coefficient of determination (*R*^2^). The formula for these metrics is presented in the following equations.

(8) $$RMSE=\sqrt{\frac{\sum_{t=1}^{N}{\left(yt-\hat{y}t\right)}^{2}}{N}}$$

(9) $$MAE=\frac{\sum_{t=1}^{N}\left|yt-\hat{y}t\right|}{N}$$

(10) $$R^{2}=\frac{\sum_{t}{\left(\hat{y}t-\bar{y}\right)}^{2}}{\sum_{t}{\left(yt-\bar{y}\right)}^{2}}$$

where yt is the actual value, $\hat{y}t$ is the predicted value, $\bar{y}$ is the mean value of *y*, and *N* is the number of observations. The evaluation metrics presented in the Results and Discussion section were calculated based on the original data. The original data was obtained by converting the normalized value with the inverse of the min–max scaling algorithm presented in Equation (7).

## 5. Case Study and Dataset Description

**Figure 6:**
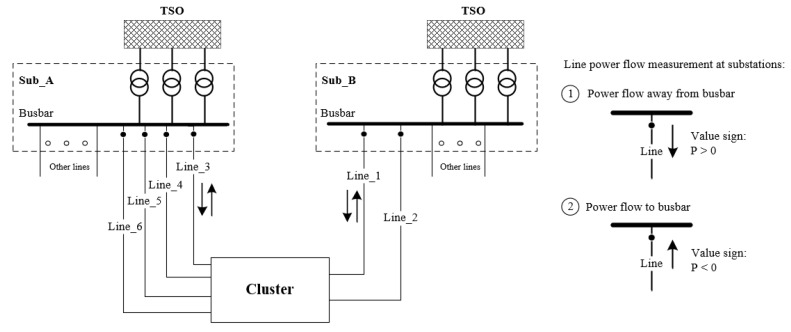
Investigated network cluster.

The complexity of the electricity system is further increased when large power plants feeding electricity into the transmission grid integrate with countless decentralized renewable energy plants feeding electricity into the medium- and low-voltage grids. This is because fluctuating electricity generation from renewable energy sources (RES) and the varying behaviour of electricity consumers lead to a change in the behaviour of the line power flow. In order to reduce the complexity of the entire power grid system and to analyse the effects of decentralized power generation from RES, a regional grid network cluster is needed. For this purpose, a simplified grid network cluster is created at the connection point between transmission system operators (TSO) and distribution system operators (DSO) using the following grid reduction procedure. First, the power grid is zoned according to the high-voltage lines and then the internal connection lines are neglected. The area between two transformer substations forms a power supply area, which is called a network cluster in this study. Within this network cluster, there are different voltage levels of loads and electricity suppliers. In this paper, one regional high-voltage subnet from northeast Germany was taken into account as an example of a network cluster. The structure of the network cluster can be seen in Figure 6.

The investigated network cluster is supplied by six feeder lines, of which four feed lines (line 3, line 4, line 5, line 6) are connected to substation A (Sub_A) and two feed lines (line 1, line 2) to substation B (Sub_B). By measuring the power of the feeder lines, we can capture the main generation and load information of the grid cluster, how much power is imported when the regional load of the grid cluster is high, and how much surplus generation is exported from the grid cluster under study. Based on the measurement of directional power, as shown in Figure 6, the sign of the power flow indicates the direction of power towards or away from the busbar. In this paper, we investigated two HDL models to predict the direction of power flow on a single line of the network under study by considering other existing lines. For example, to predict line 1 of the studied network cluster, we used the other lines (line 2, line 3, line 4, line 5, and line 6) as input references. This implementation also applies to the other lines if they are to be predicted. Since the high regional installation of renewable energy systems, about 365 MW photovoltaic systems and 630 MW wind turbines have been connected to the DSO grids studied locally. A wide range of weather data is also used in this work, as local weather has a major impact on regional electricity generation from renewable energy sources. Therefore, to find out whether weather data can influence the power flow prediction results, we included additional weather condition parameters as reference values, together with power flow values that exist on other feeder lines.

### 5.1. Bi-Directional Power Flow Measurement Data

**Figure 7:**
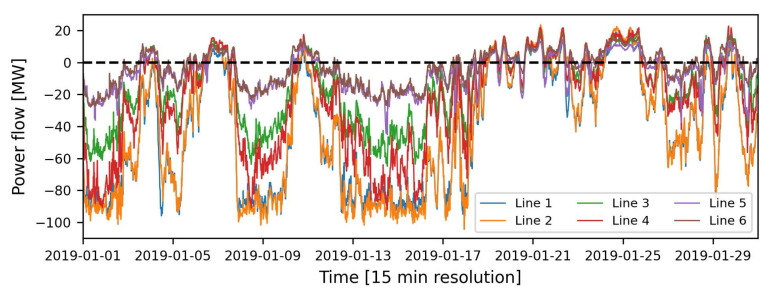
Power flow in all lines of network cluster.

To predict the direction of power flow on an individual feeder line of the investigated network cluster. We used raw directional power measurement data provided by the local distribution system operator (DSO). This directional power flow data has a temporal resolution of 15 min and ranges from 1 January 2019 to 31 December 2019. As shown in Figure 7, a value of active power above zero means that power is flowing away from the busbar to the cluster, while a value below zero means that power is flowing towards the busbar from network cluster. Figure 7 shows an example of the power flow in the network cluster studied in January 2019. The statistical description of power flow measurement dataset can be found in Table 4.

### 5.2. Weather Data

The weather data used in this paper comes from a German weather service provider. This provider offers access to the Climate Data Centre (CDC) portal to retrieve weather data with a temporal resolution of 15 min recorded by various weather measuring stations. The regional stations are filtered, and the mean weather data is calculated. The parameters of the weather data are explained in more detail below:

- Ground air temperature (2 m above ground) (°C);
- Ground wind speed (10 m above ground) (m/s);
- Solar irradiation (W/m^2^).

**Figure 8:**
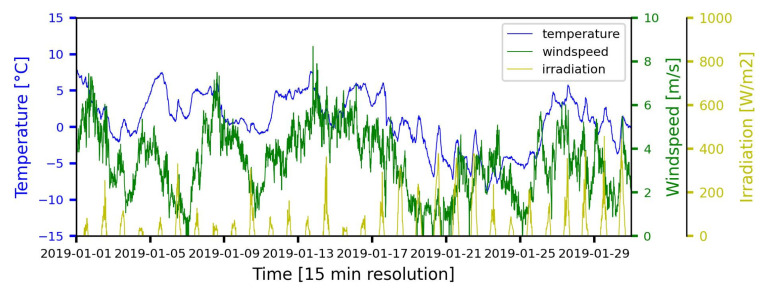
Local weather data sample.

The length of the weather data used in this paper is the same as the length of power measurement data, from 1 January 2019 to 31 December 2019. Figure 8 shows the weather conditions in the vicinity of the studied grid cluster in January 2019. The statistical description of the weather dataset used in this paper can be found in Table 5.

## 6. Result and Discussion

### 6.1. A Comparison of the Hybrid Deep Learning Model for Predicting the Direction of Power Flow of Each Line Based on Real Power Measurement Data Only

In this subsection, we present the simulation results for predicting the direction of the power flow of each feeder in the studied grid cluster, based solely on real power measurement data (dataset group 1). For this simulation, the two proposed HDL models were used together with the baseline models. To compare the performance of all the deep learning models, we considered the duration of the training period and the performance evaluation results based on various metrics, such as RMSE, MAE and R^2^. During the training period, all deep learning models were re-trained for different sub-datasets with the same fitting configurations. The reason for this is that the developed method is to predict the direction of power flow of a single line in real time based on the other existing lines of the network cluster. For example, if the DL model wants to predict the power flow in line 1, it needs reference values for the power flow of lines 2, 3, 4, 5 and 6. This implementation also applies to other lines if they are to be predicted. Therefore, six individual partial prediction models of the individual deep learning models were created for each line.

**Figure 9:**
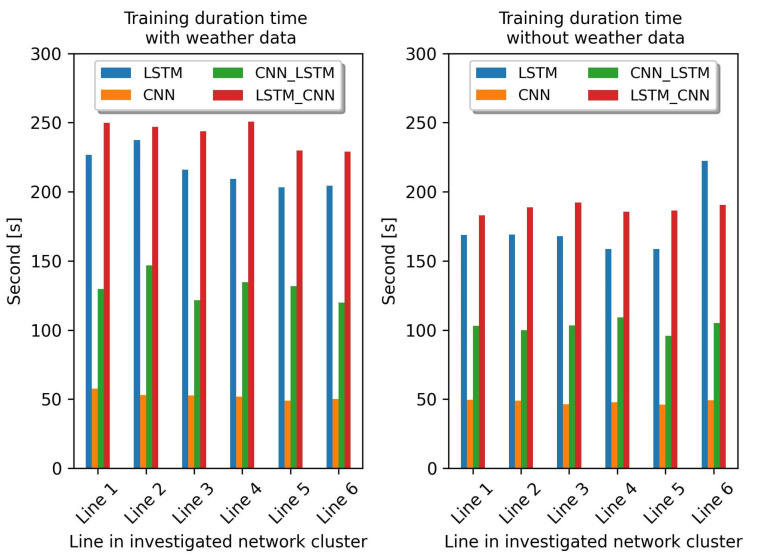
Training duration time of all the deep learning models.

As for the comparison of the training time of all the deep learning models, we can see this in Table 6. The proposed HDL model of CNN-LSTM always has shorter training times compared to the proposed model of LSTM-CNN in all the line-of-grid clusters studied, although they have similar constructed layers and parameters used. Basically, CNNs are designed to be faster because the computations in CNNs can be performed in parallel, whereas LSTMs have to be processed sequentially because the next step depends on the previous one. This can be illustrated in Figure 9 (right side), where the CNN trained with the group 1 dataset (dataset containing only power measurements) has a faster training time than other deep learning models. Therefore, the CNN network placed in the first layer of the proposed HDL model can lead to various complexity reductions by focusing on the most important features. The use of convolutional layers leads to a reduction in the size of the tensor and the use of pooling layers also leads to a further reduction. This is one of the reasons why the model CNN-LSTM can be faster than LSTM-CNN.

With regard to the comparison of assessment performances. In Table 7, we see that the two proposed HDL models are quite competitive in predicting power flow. Therefore, we also need to compare the two HDL models with the baseline models. According to the metrics RMSE, MAE, and R^2^, the LSTM-CNN model performs better than the CNN-LSTM model in predicting the power flow on lines 1, 2, 3, and 5, while the CNN-LSTM model performs better only on lines 4 and 6. However, the overall comparison with the baseline models, the metrics RMSE and R^2^ shows the LSTM model has the best performance among all the deep learning models in predicting the power flow on lines 1, 3, 4, and 5, while the proposed model LSTM-CNN has the best performance in predicting lines 2 and CNN-LSTM for line 6. For the metric MAE, the LSTM model performs better than all other models in predicting lines 3, 4, and 5, while the model LSTM-CNN performs better than all other models in predicting lines 1 and 2 and the model CNN-LSTM performs better than all other models in predicting line 6. Based on these simulation results, the HDL model does not perform better than the single model of LSTM in predicting the power flow of the network cluster under study.

**Figure 10:**
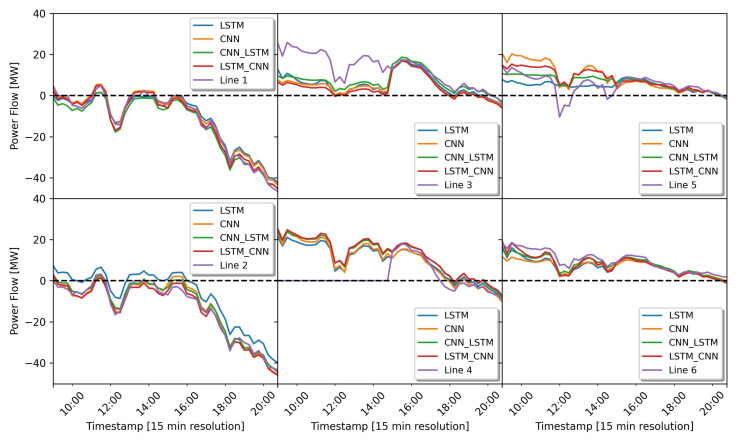
Prediction results of the power flow direction based on real power measurement data only.

In this prediction simulation, the direction of the power flow can be determined by the value of the power flow itself. If the power value is below zero, the direction of the power flow is from the network cluster to the busbar, while if it is above zero, it flows from the busbar to the network cluster. The power flow prediction results of all the deep learning models can be seen in Figure 10. This figure describes the prediction results for the test dataset covering the period from 7 November 2019 at 09:00 to 7 November 2019 at 20:45. As we can see, all deep learning models are generally equally good at following the original value of the power flow measurement (purple line). In addition, We can also see in this figure that most of the active power values (purple lines) on the feeder lines 3 and 4 are always above 0. This indicates that the direction of power flow during this period tends to be towards the grid cluster. On feeder lines 1 and 2, on the other hand, the power flow is always below zero, indicating that there is a power surplus from the grid cluster and the power flow is directly to the busbar. However, on closer inspection, the original power flow pattern (purple line) on lines 1 and 2 are similar because these lines are connected in parallel. Line 3 is also connected in parallel with line 4, as is line 5 to line 6.

### 6.2. A Comparison of the Hybrid Deep Learning Model for Predicting the Direction of Power Flow of Each Line Based on Real Power Measurement Data and Local Weather Data

In this sub-section, we show the simulation and test results of the proposed HDL model for power flow prediction based on the input dataset containing power values along with weather parameters (dataset group 2). The procedure used in the simulation to predict the direction of power flow on the single feeder is exactly the same as in the previous subsection. The only difference is the dataset used, as the purpose of this simulation was to determine the extent to which weather data can affect the power flow prediction results. Thus, if the model wants to predict the power flow in line 1, it needs reference values for the power flow of lines 2, 3, 4, 5 and 6, as well as three additional weather parameters, including air temperature, wind speed and solar irradiation

The competition between the CNN-LSTM and LSTM-CNN is also quite close in this simulation, as Table 8 shows. Judging by the comparison of the training time (see Table 6), the model CNN-LSTM always has a rather short training time compared to LSTM-CNN. This is also evidenced by the training time in the previous section. Therefore, it can be assumed that the training time of the CNN-LSTM model is indeed faster than that of the LSTM-CNN model. However, the CNN model still has the fastest training time compared to all models of DL (see Figure 9, left side). As for the comparison of the datasets used, Figure 9 shows in detail that the training time of all models becomes longer when the training datasets becomes larger. This can be seen when the input dataset for training consists of power flow and local weather data. All models tend to have a longer training time than the input dataset consisting only of power flow data. From this experiment, it can be concluded that the size of the input dataset is a factor that significantly influences the duration of the model training time.

From a performance comparison perspective in this simulation, the CNN-LSTM and LSTM-CNN models tend to have the same ability to predict power flow. According to the metrics RMSE, MAE and R^2^, LSTM-CNN performs very well in predicting power flow in lines 1 and 2, while CNN-LSTM surprisingly performs better in predicting lines 3, 4, 5 and 6. Since the proposed HDL model has similar performance, we considered the LSTM only and the CNN only as the baseline models in this simulation and compared them with the proposed HDL model. The experimental results (see Table 8) show the HDL model performs much better than the single model. Based on the MAE metric, LSTM-CNN performs better in predicting lines 1, 2, and 3 compared to the other models. Meanwhile, CNN-LSTM is excellent in predicting lines 4 and 6 compared to the other models, and the last one, the LSTM model, is the only good at predicting line 5. From the RMSE and R^2^ perspective, the LSTM-CNN performs better compared to the other models in predicting the power flow at lines 1 and 2, while CNN-LSTM is outstanding in predicting line 6 only. Unexpectedly, the CNN model performs well in predicting lines 3 and 4 compared to the other models. Meanwhile in line 5, only LSTM has the best performance to predict power flow.

**Figure 11:**
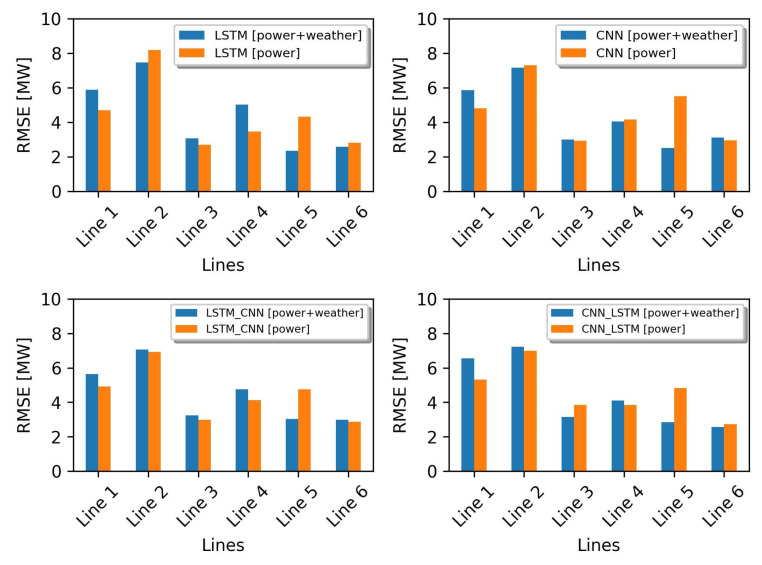
Performance comparison based on the RMSE metric.

**Figure 12:**
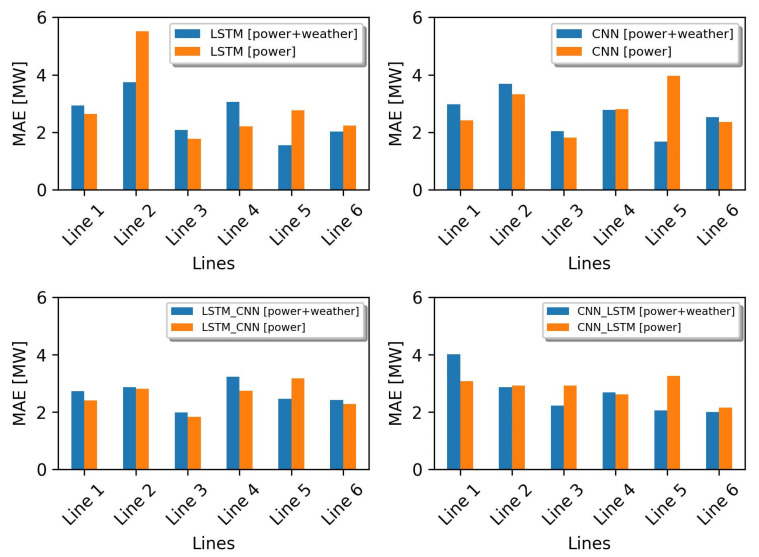
Performance comparison based on the MAE metric.

**Figure 13:**
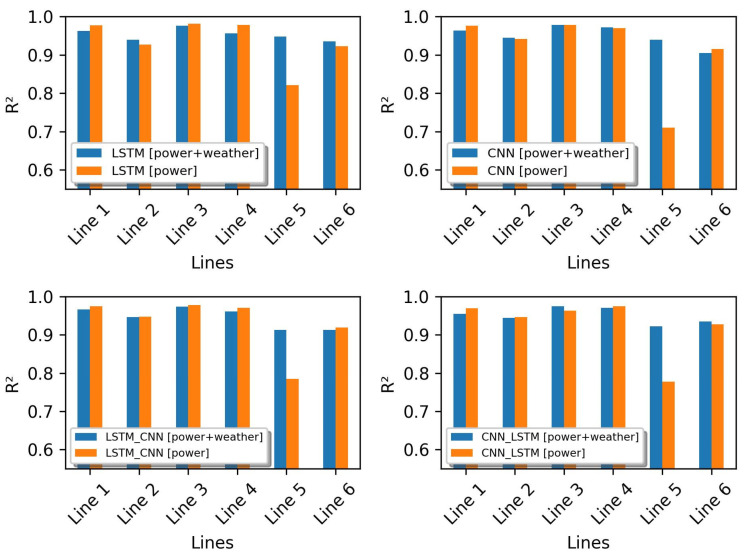
Performance comparison based on the R^2^ metric.

From the experiments conducted, the performance of the proposed HDL model and the baseline models tend to differ when two different datasets are used. In the previous simulation, all models were trained with the dataset of group 1 (dataset containing only power flow data). The evaluation results show that the HDL model is not superior to the baseline models. In contrast, the performance of the HDL model is good compared to the baseline models in the simulation where all models were trained with the datasets of group 2 (containing power flow and weather parameters). Using these simulation results (see Table 7 and Table 8), we can check the performance of each model when trained with two different datasets in terms of size and input parameters. The results of the performance comparison between the models trained with the dataset of group 1 (orange bar) and the models trained with the dataset of group 2 (blue bar) can be seen in Figure 11, Figure 12 and Figure 13. From these figures, it can be seen that all the evaluation metrics (RMSE, MAE, and R^2^) show that the addition of weather parameters to the training dataset can affect the performance of all the models but does not have a significant impact. On closer inspection, the weather data can improve the performance of the LSTM in predicting the power flow in lines 2, 5, and 6. While the CNN model has a better performance in lines 2, 4, and 5.

For the HDL model, adding weather parameters to the dataset used in the training phase has a good impact on model performance. On closer inspection (see Figure 11, Figure 12 and Figure 13), the evaluation results show that the model LSTM-CNN with additional weather parameters gives a better prediction of the power flow in lines 2, 3, 5 and 6. In contrast, the CNN-LSTM model only has an effect on line 5.

**Figure 14:**
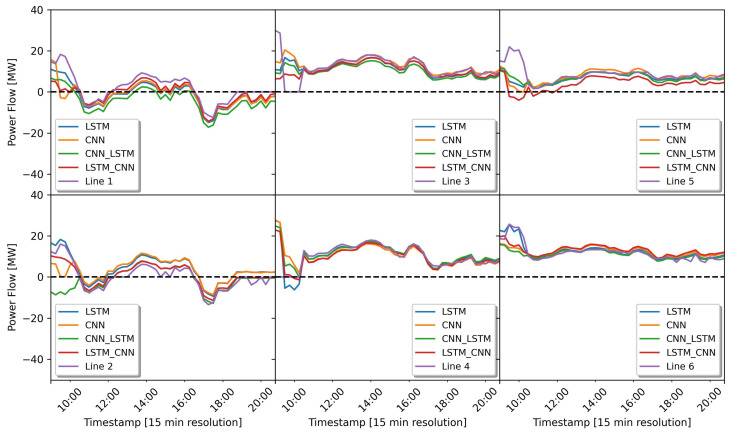
Prediction results of the power flow direction based on real power measurement and weather data.

The results of predicting the power flow is very important in determining its direction. In this sub-section. The results of predicting the power flow of all the deep learning models trained with the dataset of group 2 (the dataset containing power values and weather parameters) is shown in Figure 14. The test dataset used in this simulation covers the period from 8 November 2019 at 09:00 to 8 November 2019 at 20:45. Looking closely at the prediction results, all the trained models are generally equally good at following the original value of the power flow measurement (purple line). As we can see from feeder lines 3, 4, 5 and 6 (see Figure 14). The power flow is always above zero, which indicates that the direction of the power flow is from the busbar to the cluster grid. This is because there is a high load demand in the grid cluster.

## 7. Conclusions

As the growth and deployment of renewable energy systems increases rapidly, the energy exchange becomes more complex. This is because consumers act as prosumers, meaning they have the ability to generate electricity and synchronize with the grid. They also tend to have different types of loads that are predominantly inductive or capacitive. This phenomenon will draw active energy from the system or towards the system. Therefore, it is necessary to use the correct method to determine exactly how much active and reactive energy is generated in the power system under export or import conditions. Knowing the direction of power flow during energy exchange is very important for distribution companies as it can help control the energy generated or distributed during the energy exchange between different but interconnected utilities.

In this study, two of the most popular hybrid deep learning models were proposed and compared with the baseline models (CNN only and LSTM only) to predict the direction of power flow on each line of a network cluster. To compare the performance of all the deep learning models, we have considered the duration of the training period and the performance evaluation results based on various metrics, such as RMSE, MAE and R^2^. In the training process, two types of datasets were used to test the capability of the proposed HDL model. The first type was a dataset containing real power measurement data, and the second type was a group of datasets containing real power measurement and local weather data. The purpose of dividing this group of datasets was to see if the size of the dataset and the weather data affected the performance of the proposed model.

The experimental results show that the weather parameters in the dataset can increase the size of the datasets and increase the training time of all the models. Therefore, the size of the input dataset may affect the training time of the model. In terms of performance evaluation, the proposed HDL model did not perform better than the baseline models in predicting the power flow of the studied grid cluster when trained with the power flow data only. However, in contrast, the proposed HDL models showed good performance compared to the baseline models when trained with a dataset that included additional weather parameters. In this study, the metric evaluation of RMSE, MAE and R^2^ shows that both proposed HDL models can reach a small error under certain conditions, so it is still relatively challenging to determine the best HDL model for predicting the direction of power flow in the investigated network cluster.

## Figures and Tables

**Table 1:** Summary of related work.

Topic	Reference	Methodology/Output
Prediction on power flow	Brauns et al. [3]	Using LSTM model for vertical power flow forecasting.
	Park et al. [22]	Implementing machine learning for predicting adaptive power flow.
	Schafer et al. [23]	Proposed ANN with data pre-processing techniques to solve power flow prediction.
	Our	Proposed CNN-LSTM and LSTM-CNN models for bi-directional power flow prediction.
CNN-LSTM and LSTM-CNN for prediction	Song et al. [24]	CNN-LSTM performs excellently in the area of predictive accuracy.
	Agga et al. [25]	CNN-LSTM performs better than the standard ML or individual DL.
	Farsi et al. [26]	Parallel LSTM-CNN is a good candidate for use as a short-term prediction tool.
	Li et al. [27]	Parallel LSTM-CNN has a good prediction effect.

**Table 2:** Hybrid deep learning model structure.

HDL Model	Structure of Layer
LSTM	LSTM layer (neurons: 30) + LSTM layer (neurons: 15) + dense layer (neuron: 1, LeakyRelu activation)
CNN	conv1D layer (filters: 16, filter size: 3, relu activation) + conv1D layer (filters: 16, filter size: 3, relu activation) + maxpooling1D (polling size: 2, padding: same) + flatten layer+ dense layer (neuron: 1, LeakyRelu activation)
CNN-LSTM	Conv1D layer (filters: 32, filter size: 3, relu activation) + conv1D layer (filters: 32, filter size: 3, relu activation) + maxpooling1D (polling size: 2, padding:same) + flatten layer + LSTM layer (neurons: 32, relu activation) + LSTM layer (neurons: 10) + dense layer (neuron: 1, LeakyRelu activation)
LSTM-CNN	LSTM layer (neurons: 32, relu activation) + LSTM layer (neurons: 10) + conv1D layer (filters: 32, filter size: 3, relu activation) + conv1D layer (filters: 32, filter size: 3, relu activation) + maxpooling1D (polling size: 2, padding: same) + flatten layer+ dense layer (neuron: 1, LeakyRelu activation)

**Table 3:** Machine specification.

Parameter	Specification
CPU	Intel(R) Core(TM) i5-7200U CPU @ 2.50 GHz 2.71 GHz
GPU	Intel UHD Graphics 620
HDD/SDD	750 GB
RAM	16 GB
OS	Windows 10 pro 64-bit

**Table 4:** Statistical description of the power flow measurement data.

Feed Lines	Power Flow Away from Busbar				Power Flow to Busbar			
	Number of Data Point	Min [MW]	Mean [MW]	Max [MW]	Number of Data Point	Min [MW]	Mean [MW]	Max [MW]
Line 1	3287	2.378	7.472	22.189	29323	−104.809	−41.40	−2.378
Line 2	3434	2.378	7.544	23.559	29073	−104.417	−41.19	−2.378
Line 3	11843	0.878	9.162	34.311	21422	−102.501	−21.030	−1.095
Line 4	10574	0.878	9.536	25.230	22760	−115.122	−25.798	−1.095
Line 5	12377	0.878	5.694	21.919	19951	−74.392	−15.507	−1.095
Line 6	15467	0.878	7.072	25.676	16918	−48.257	−10.656	−1.095

**Table 5:** Statistical descriptive of local weather data.

Weather Parameter	Number of Data Point	Min	Mean	Max
Temperature	34,826	−9.02	11.23	37.31
Windspeed	34,826	0.00	2.59	10.39
Irradiation	34,826	0.00	135.33	1310.83

**Table 6:** Training time of the deep learning models.

Dataset	DL Model	Training Time (Second)					
		Line 1	Line 2	Line 3	Line 4	Line 5	Line 6
Power data	LSTM	168.849	169.043	167.98	158.55	158.56	222.20
	CNN	49.522	48.855	46.316	47.85	46.11	49.22
	CNN-LSTM	102.955	99.872	103.379	109.088	95.847	105.188
	LSTM-CNN	182.87	188.738	192.276	185.572	186.291	190.605
Power + Weather data	LSTM	226.62	237.25	215.84	209.41	203.17	204.47
	CNN	57.76	52.95	52.60	51.76	49.07	50.24
	CNN-LSTM	129.591	146.837	121.648	134.741	131.83	119.886
	LSTM-CNN	249.918	246.845	243.63	250.825	229.769	228.971

**Table 7:** Performance evaluation of the deep learning models based on power flow data.

Metric Evaluation	DL Model	Line Predicted					
		Line 1	Line 2	Line 3	Line 4	Line 5	Line 6
RMSE (MW)	LSTM	4.694	8.195	2.698	3.47	4.33	2.81
	CNN	4.823	7.299	2.931	4.16	5.51	2.94
	CNN-LSTM	5.31	6.986	3.84	3.85	4.815	2.725
	LSTM-CNN	4.922	6.917	2.991	4.118	4.747	2.877
MAE (MW)	LSTM	2.643	5.521	1.766	2.21	2.76	2.23
	CNN	2.412	3.318	1.811	2.81	3.97	2.36
	CNN-LSTM	3.078	2.917	2.921	2.613	3.265	2.152
	LSTM-CNN	2.408	2.809	1.838	2.745	3.175	2.281
R^2^	LSTM	0.977	0.927	0.982	0.98	0.82	0.92
	CNN	0.976	0.942	0.978	0.97	0.71	0.92
	CNN-LSTM	0.97	0.947	0.963	0.975	0.778	0.93
	LSTM-CNN	0.975	0.948	0.978	0.971	0.785	0.92

**Table 8:** Performance evaluation of the deep learning models based on the power flow and weather data.

Metric Evaluation	DL Model	Line Predicted					
		Line 1	Line 2	Line 3	Line 4	Line 5	Line 6
RMSE (MW)	LSTM	5.897	7.475	3.075	5.026	2.335	2.589
	CNN	5.858	7.169	2.992	4.042	2.51	3.124
	CNN-LSTM	6.553	7.232	3.149	4.095	2.849	2.573
	LSTM-CNN	5.649	7.056	3.242	4.746	3.026	2.98
MAE (MW)	LSTM	2.936	3.735	2.074	3.056	1.552	2.021
	CNN	2.974	3.689	2.036	2.782	1.673	2.526
	CNN-LSTM	4.015	2.871	2.231	2.686	2.052	2.006
	LSTM-CNN	2.733	2.868	1.985	3.23	2.468	2.416
R^2^	LSTM	0.963	0.94	0.976	0.957	0.948	0.935
	CNN	0.964	0.945	0.978	0.972	0.94	0.905
	CNN-LSTM	0.955	0.944	0.975	0.971	0.922	0.935
	LSTM-CNN	0.966	0.946	0.974	0.961	0.913	0.913

## Data Availability

The data are not publicly available due to the policy of the associate company.

## References

[1] Wang Q., Dong Z., Li R., Wang L. (2022). Renewable energy and economic growth: New insight from country risks. Energy.

[2] Aslam M., Lee J.-M., Kim H.-S., Lee S.-J., Hong S. (2019). Deep Learning Models for Long-Term Solar Radiation Forecasting Considering Microgrid Installation: A Comparative Study. Energies.

[3] Brauns K., Scholz C., Schultz A., Baier A., Jost D. (2021). Vertical power flow forecast with LSTMs using regular training update strategies. Energy AI.

[4] Li Y., Janik P., Schwarz H., Pfeiffer K. (2022). Proposal of a regional grid cluster model for analysis of electrical power net-work performance. Arch. Electr. Eng..

[5] Suresh G., Prasad D., Gopila M. (2021). An efficient approach based power flow management in smart grid system with hybrid renewable energy sources. Renew. Energy Focus.

[6] Aslam S., Herodotou H., Mohsin S.M., Javaid N., Ashraf N., Aslam S. (2021). A survey on deep learning methods for power load and renewable energy forecasting in smart microgrids. Renew. Sustain. Energy Rev..

[7] Zhou H., Wang S., Miao Z., He C., Liu S. (2019). Review of The Application of Deep Learning in Fault Diagnosis. Chin. Control Conf. CCC.

[8] Aksan F., Janik P., Suresh V., Leonowicz Z. Review of the application of deep learning for fault detection in wind turbine. Proceedings of the 2022 IEEE International Conference on Environment and Electrical Engineering and 2022 IEEE Industrial and Commercial Power Systems Europe (EEEIC/I&CPS Europe).

[9] Alkhayat G., Mehmood R. (2021). A review and taxonomy of wind and solar energy forecasting methods based on deep learning. Energy AI.

[10] Cecaj A., Lippi M., Mamei M., Zambonelli F. (2020). Comparing Deep Learning and Statistical Methods in Forecasting Crowd Distribution from Aggregated Mobile Phone Data. Appl. Sci..

[11] Fallah S.N., Deo R.C., Shojafar M., Conti M., Shamshirband S. (2018). Computational Intelligence Approaches for Energy Load Forecasting in Smart Energy Management Grids: State of the Art, Future Challenges, and Research Directions. Energies.

[12] Wu Y.-X., Wu Q.-B., Zhu J.-Q. (2019). Data-driven wind speed forecasting using deep feature extraction and LSTM. IET Renew. Power Gener..

[13] Yu C., Li Y., Bao Y., Tang H., Zhai G. (2018). A novel framework for wind speed prediction based on recurrent neural networks and support vector machine. Energy Convers. Manag..

[14] Tong X., Wang J., Zhang C., Wu T., Wang H., Wang Y. (2022). LS-LSTM-AE: Power load forecasting via Long-Short series features and LSTM-Autoencoder. Energy Rep..

[15] Wang F., Xuan Z., Zhen Z., Li K., Wang T., Shi M. (2020). A day-ahead PV power forecasting method based on LSTM-RNN model and time correlation modification under partial daily pattern prediction framework. Energy Convers. Manag..

[16] Suresh V., Aksan F., Janik P., Sikorski T., Revathi B.S. (2022). Probabilistic LSTM-Autoencoder Based Hour-Ahead Solar Power Forecasting Model for Intra-Day Electricity Market Participation: A Polish Case Study. IEEE Access.

[17] Kumar S., Hussain L., Banarjee S., Reza M. Energy Load Forecasting using Deep Learning Approach-LSTM and GRU in Spark Cluster. Proceedings of the 2018 Fifth International Conference on Emerging Applications of Information Technology (EAIT).

[18] Feng C., Zhang J. (2020). SolarNet: A sky image-based deep convolutional neural network for intra-hour solar forecasting. Sol. Energy.

[19] Suresh V., Janik P., Rezmer J., Leonowicz Z. (2020). Forecasting Solar PV Output Using Convolutional Neural Networks with a Sliding Window Algorithm. Energies.

[20] Fu J., Chu J., Guo P., Chen Z. (2019). Condition Monitoring of Wind Turbine Gearbox Bearing Based on Deep Learning Model. IEEE Access.

[21] Zang H., Liu L., Sun L., Cheng L., Wei Z., Sun G. (2020). Short-term global horizontal irradiance forecasting based on a hybrid CNN-LSTM model with spatiotemporal correlations. Renew. Energy.

[22] Park J., Kodaira D., Agyeman K., Jyung T., Han S. (2021). Adaptive Power Flow Prediction Based on Machine Learning. Energies.

[23] Schäfer F., Menke J.-H., Braun M. (2020). Prediction of power flow results in time-series-based planning with artificial neural networks and data pre-processing. CIRED—Open Access Proc. J..

[24] Song J., Zhang L., Xue G., Ma Y., Gao S., Jiang Q. (2021). Predicting hourly heating load in a district heating system based on a hybrid CNN-LSTM model. Energy Build..

[25] Agga A., Abbou A., Labbadi M., El Houm Y., Ali I.H.O. (2022). CNN-LSTM: An efficient hybrid deep learning architecture for predicting short-term photovoltaic power production. Electr. Power Syst. Res..

[26] Farsi B., Amayri M., Bouguila N., Eicker U. (2021). On Short-Term Load Forecasting Using Machine Learning Techniques and a Novel Parallel Deep LSTM-CNN Approach. IEEE Access.

[27] Li C., Hu R., Hsu C.-Y., Han Y. Short-term Power Load Forecasting based on Feature Fusion of Parallel LSTM-CNN. Proceedings of the 2022 IEEE 4th International Conference on Power, Intelligent Computing and Systems (ICPICS).

[28] Hochreiter S., Schmidhuber J. (1997). Long short-term memory. Neural Comput..

[29] Tulensalo J., Seppänen J., Ilin A. (2020). An LSTM model for power grid loss prediction. Electr. Power Syst. Res..

[30] Qing X., Niu Y. (2018). Hourly day-ahead solar irradiance prediction using weather forecasts by LSTM. Energy.

[31] Ghosh A., Sufian A., Sultana F., Chakrabarti A., De D. (2019). Fundamental Concepts of Convolutional Neural Network. Intell. Syst. Ref. Libr..

[32] Habeck C., Gazes Y., Razlighi Q., Stern Y. (2020). Cortical thickness and its associations with age, total cognition and education across the adult lifespan. PLoS ONE.

[33] Mishra B., Shahi T.B. (2021). Deep learning-based framework for spatiotemporal data fusion: An instance of Landsat 8 and Sentinel 2 NDVI. J. Appl. Remote. Sens..

[34] Plakias S., Boutalis Y.S. (2020). Fault detection and identification of rolling element bearings with Attentive Dense CNN. Neurocomputing.

[35] Chen L., Xu G., Zhang Q., Zhang X. (2019). Learning deep representation of imbalanced SCADA data for fault detection of wind turbines. Measurement.

[36] Aksan F., Jasiński M., Sikorski T., Kaczorowska D., Rezmer J., Suresh V., Leonowicz Z., Kostyła P., Szymańda J., Janik P. (2021). Clustering Methods for Power Quality Measurements in Virtual Power Plant. Energies.

[37] Lee T., Singh V.P., Cho K.H. (2021). Deep Learning for Time Series. Water Sci. Technol. Libr..

[38] Wang K., Qi X., Liu H. (2019). A comparison of day-ahead photovoltaic power forecasting models based on deep learning neural network. Appl. Energy.

[39] Wang K., Qi X., Liu H. (2019). Photovoltaic power forecasting based LSTM-Convolutional Network. Energy.

[40] Memarzadeh G., Keynia F. (2020). A new short-term wind speed forecasting method based on fine-tuned LSTM neural network and optimal input sets. Energy Convers. Manag..

[41] Rahimilarki R., Gao Z., Jin N., Zhang A. (2019). Time-series Deep Learning Fault Detection with the Application of Wind Turbine Benchmark. IEEE Int. Conf. Ind. Inform..

[42] Lu X., Lin P., Cheng S., Lin Y., Chen Z., Wu L., Zheng Q. (2019). Fault diagnosis for photovoltaic array based on convolutional neural network and electrical time series graph. Energy Convers. Manag..

[43] Hwang H.P.-C., Ku C.C.-Y., Chan J.C.-C. (2021). Detection of Malfunctioning Photovoltaic Modules Based on Machine Learning Algorithms. IEEE Access.

[44] Jahangir H., Golkar M.A., Alhameli F., Mazouz A., Ahmadian A., Elkamel A. (2020). Short-term wind speed forecasting framework based on stacked denoising auto-encoders with rough ANN. Sustain. Energy Technol. Assessments.

[45] Hong Y.-Y., Rioflorido C.L.P.P. (2019). A hybrid deep learning-based neural network for 24-h ahead wind power forecasting. Appl. Energy.

